# Endotype-Phenotype Patterns in Meniere's Disease Based on Gadolinium-Enhanced MRI of the Vestibular Aqueduct

**DOI:** 10.3389/fneur.2019.00303

**Published:** 2019-04-05

**Authors:** David Bächinger, Catrin Brühlmann, Tim Honegger, Eleftheria Michalopoulou, Arianne Monge Naldi, Vincent G. Wettstein, Stefanie Muff, Bernhard Schuknecht, Andreas H. Eckhard

**Affiliations:** ^1^Department of Otorhinolaryngology, Head and Neck Surgery, University Hospital Zurich, Zurich, Switzerland; ^2^University of Zurich, Zurich, Switzerland; ^3^Department of Biostatistics, Institute for Epidemiology, Biostatistics, and Prevention, University of Zurich, Zurich, Switzerland; ^4^Department of Otorhinolaryngology, University Children's Hospital Zurich, Zurich, Switzerland; ^5^Medical Radiological Institute (MRI), Zurich, Switzerland

**Keywords:** endolymphatic sac, patient subgroups, phenotype, degeneration, hypoplasia

## Abstract

Two histopathological subtypes of Meniere's disease (MD) were recently described in a human post-mortem pathology study. The first subtype demonstrated a degenerating distal endolymphatic sac (ES) in the affected inner ear (subtype MD-dg); the second subtype (MD-hp) demonstrated an ES that was developmentally hypoplastic. The two subtypes were associated with different clinical disease features (phenotypes), suggesting that distinct endotype-phenotype patterns exist among MD patients. Therefore, clinical endotyping based on ES pathology may reveal clinically meaningful MD patient subgroups. Here, we retrospectively determined the ES pathologies of clinical MD patients (*n* = 72) who underwent intravenous delayed gadolinium-enhanced inner ear magnetic resonance imaging using previously established indirect radiographic markers for both ES pathologies. Phenotypic subgroup differences were evidenced; for example, the MD-dg group presented a higher average of vertigo attacks (ratio of vertigo patterns daily/weekly/other vs. monthly, MD-dg: 6.87: 1; MD-hp: 1.43: 1; *p* = 0.048) and more severely reduced vestibular function upon caloric testing (average caloric asymmetry ratio, MD-dg: 30.2% ± 30.4%; MD-hp: 13.5% ± 15.2%; *p* = 0.009), while the MD-hp group presented a predominantly male sex ratio (MD-hp: 0.06:1 [f/m]; MD-dg: 1.2:1 [f/m]; *p* = 0.0004), higher frequencies of bilateral clinical affection (MD-hp: 29.4%; MD-dg: 5.5%; *p* = 0.015), a positive family history for hearing loss/vertigo/MD (MD-hp: 41.2%; MD-dg: 15.7%; *p* = 0.028), and radiographic signs of concomitant temporal bone abnormalities, i.e., semicircular canal dehiscence (MD-hp: 29.4%; MD-dg: 3.6%; *p* = 0.007). In conclusion, this new endotyping approach may potentially improve the diagnosis, prognosis and clinical decision-making for individual MD patients.

## Introduction

A major challenge in diagnosing and managing patients with Meniere's disease (MD) is its heterogeneous clinical presentation (i.e., phenotypic differences among patients) (1). For example, vestibular and auditory symptoms may occur together or in a temporally independent manner, in varying frequency patterns (e.g., daily, monthly, episodically), and with some symptoms predominating over others (e.g., more pronounced vestibular than auditory symptoms) (2, 3). Symptom attacks may occur in response to identifiable triggers [e.g., high sodium intake, caffeine, stress, reviewed in (1, 4)] or in random patterns. In many cases, the disease remains unilateral (one inner ear clinically affected), whereas progression to bilateral disease is observed in ~40% of cases (2, 5). In the long-term, the degree of permanent hearing loss and vestibular hypofunction can remain mild to moderate or progress to severe levels (2). Identifying distinct phenotypic patterns among MD patients would potentially enable clinicians to predict clinical features or the disease course in individual MD patients.

In a recent post-mortem histopathology study on inner ear tissues from MD patients (6), we identified two histopathological subtypes of the disease: (i) epithelial degeneration of the endolymphatic sac (ES) and (ii) organ hypoplasia of the ES. Both ES pathologies result in loss of the normal ES epithelium and its ion transport functions. Loss of ES function presumably underlies the endolymphatic hydrops generation in MD and is believed to be a necessary precondition for MD's clinical symptomatology. We therefore consider the two ES pathologies to be etiopathologically distinct MD “endotypes” that may be associated with different phenotypical features. To investigate endotype-phenotype patterns in clinical patients, we previously developed a high-resolution computed tomography (HRCT)-based imaging method to distinguish the two ES pathologies (7).

The present study aimed to (i) adapt the method to distinguish ES pathologies based on gadolinium-enhanced magnetic resonance imaging (Gd-MRI); (ii) retrospectively subgroup a series of MD patients based on their ES pathology; and (iii) investigate, based on clinical data, whether these endotypes separate into clinically meaningful patient subgroups.

## Methods

### Study Design and Approval

This retrospective explorative study was approved by the local Ethics Committee (application KEK-ZH-Nr. 2016-01619, Kantonale Ethikkommission, Zurich, Switzerland) in accordance with the Helsinki declaration and its amendments. Written informed general consent was obtained from all participants.

### Study Population

**Figure 1:**
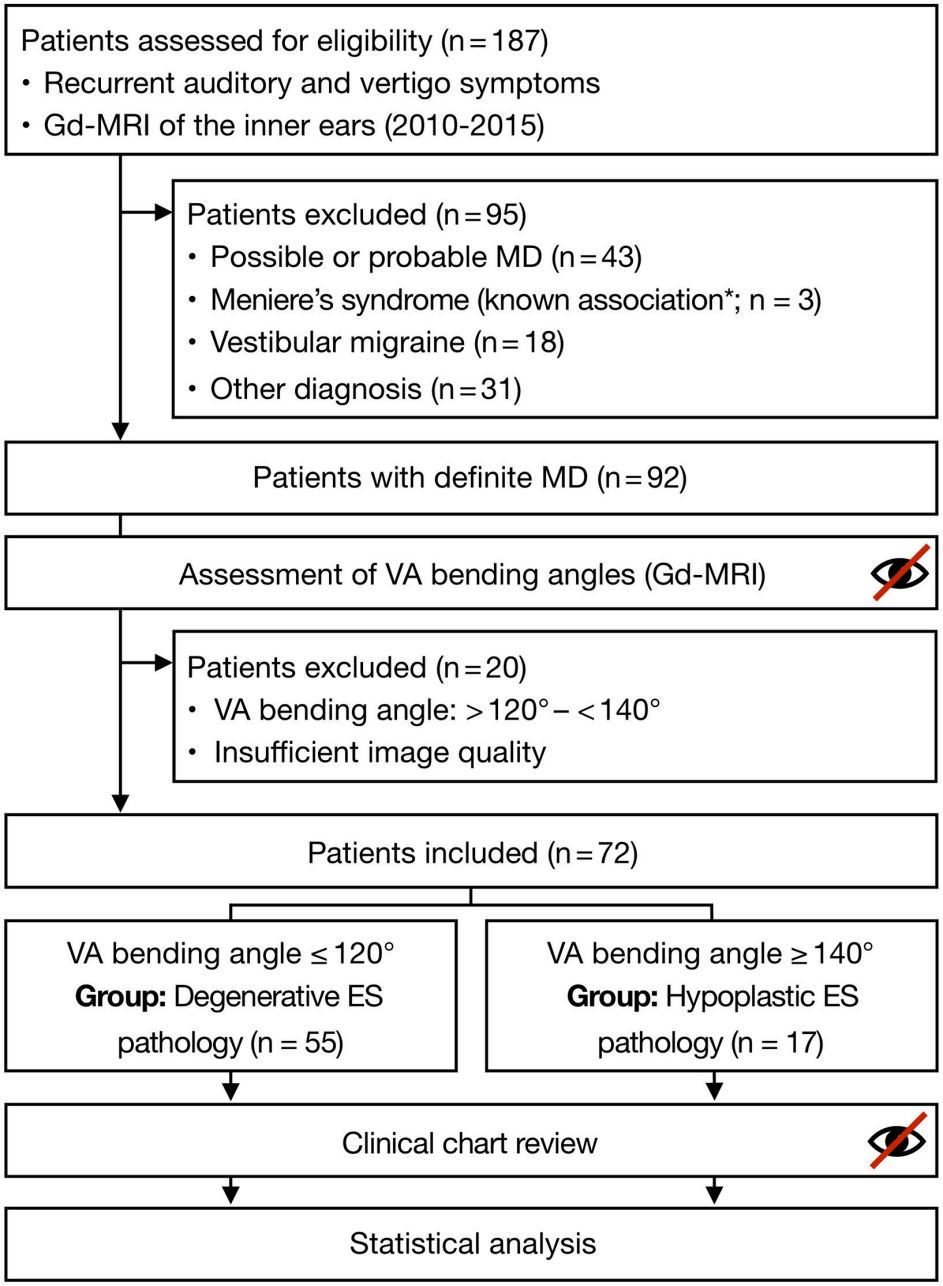
Study flowchart. MD was definitively diagnosed per the guidelines from the Committee on Hearing and Equilibrium of the American Academy of Otolaryngology-Head and Neck Surgery for the Definition of Meniere's Disease from 1995 (9). VA bending angles and clinical chart reviews were assessed in a blinded manner (see text for details). ^*^according to Merchant and Nadol (10). (Gd-MRI, gadolinium-enhanced magnetic resonance imaging; ES, endolymphatic sac; MD, Meniere's disease; VA, vestibular aqueduct).

We retrospectively assessed consecutive patients who attended our interdisciplinary center for vertigo and balance disorders at the University Hospital Zurich (tertiary referral center) from January 2010 to December 2015 for eligibility to be included in the study. The inclusion criteria were available data from Gd-MRI of the inner ears [(8); *n* = 187] and a final diagnosis of uni or bilateral definite MD [(9); *n* = 92]. We excluded patients with a Meniere's-like symptom complex due to a secondary pathology [e.g., otosclerosis, vestibular schwannoma, history of temporal bone fracture, and Cogan's syndrome, reviewed in (10)], patients who fulfilled the diagnostic criteria for possible or probable MD (9), and patients who were ultimately assigned another vertigo-associated diagnosis (e.g., vestibular migraine). Of the 92 primarily included MD patients, 20 were excluded after Gd-MRI data analysis (see “Vestibular aqueducts (VA) measurements and patient endotyping”). Finally, 72 patients' data were statistically analyzed (Figure 1).

### Temporal Bone Imaging

During the routine clinical work-up, patients underwent 3-T MR imaging of the temporal bones 4 h after intravenous contrast (Gd) was administered to detect endolymphatic hydrops based on previously established protocols (8, 11). The endolymphatic hydrops grade (8) and signs of semicircular canal (SCC) dehiscence (i.e., absence of low-signal bone margins around the SCCs) were evaluated by experienced neuroradiologists at the time the images were taken. Additional dedicated temporal bone HRCT imaging was available for a subset of patients (*n* = 16). HRCT data were reconstructed separately for each temporal bone in the axial plane using a standard bone algorithm.

### Vestibular Aqueduct (VA) Measurements and Patient Endotyping

**Figure 2:**
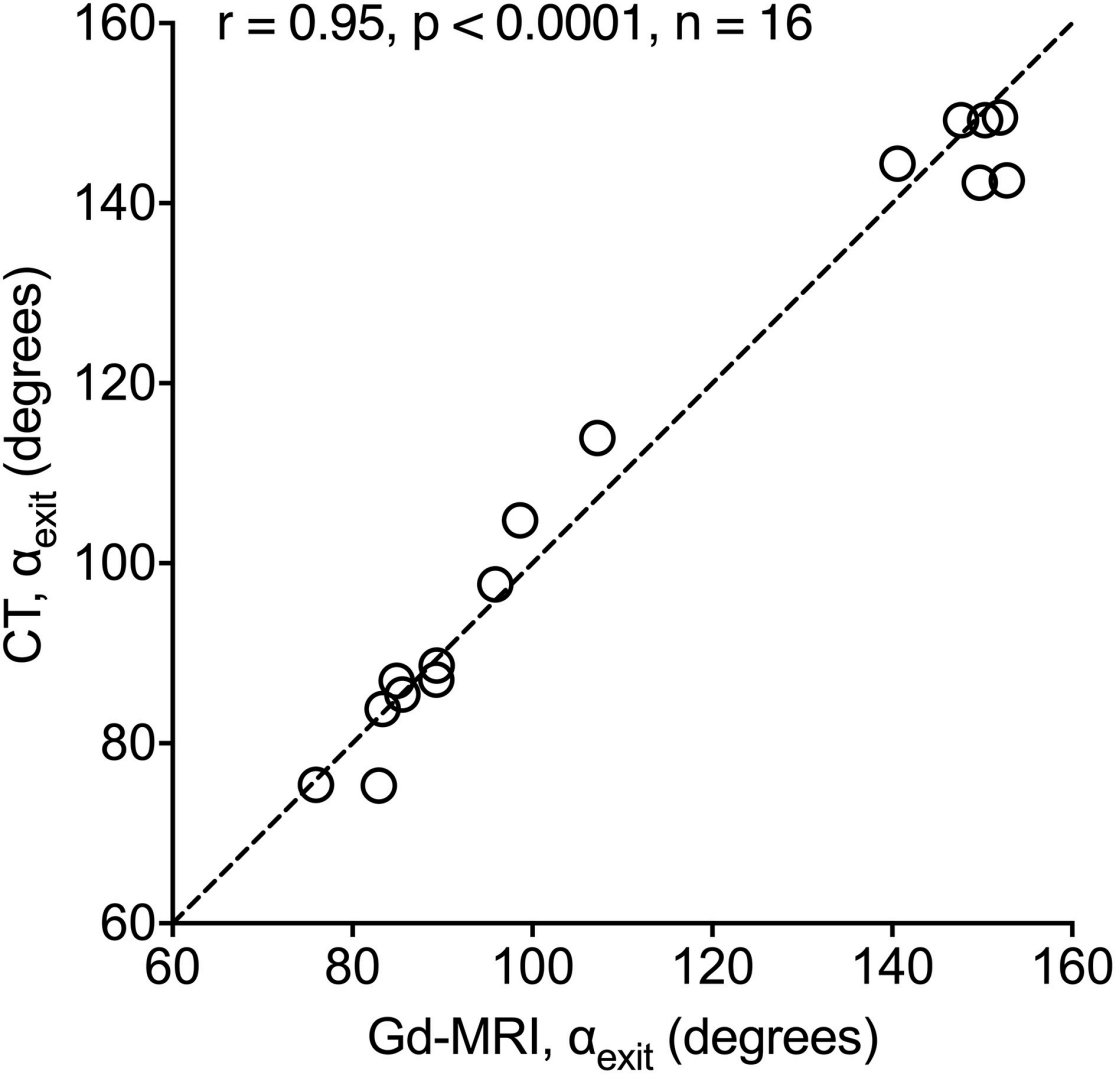
CT vs. Gd-MRI correlation for α_exit_. Angles were measured from HRCT and Gd-MRI data from the same 16 MD patients.

We recently established a method to clinically distinguish the two histopathological subtypes (ES degeneration vs. ES hypoplasia) using the vestibular aqueduct angular trajectory (ATVA) as a radiographic surrogate marker (7). We demonstrated that the two ES pathologies are associated with significantly different VA bending angles (α_exit_), which can be reliably determined using temporal bone HRCT imaging data and custom-developed software (12). Here, we validated the method for use with the Gd-MRI data by performing α_exit_ measurements based on HRCT and Gd-MRI (T2 space or 3D inversion recovery sequence) data from the same MD patients (*n* = 16). The imaging datasets were presented to an investigator (DB) who performed all measurements in a randomized order. The measured angles revealed a strong intermodal correlation between the HRCT and Gd-MRI (Spearman correlation coefficient *r* = 0.95, *p* < 0.0001; Figure 2).

Previously determined reference values for α_exit_ were used to endotype the MD patients (7). In clinically affected inner ears, angles ≤120° indicated a degenerative ES pathology. This subgroup is hereafter referred to as “MD-dg” patients. Angles ≥140° indicated a hypoplastic ES pathology, hereafter referred to as “MD-hp” patients. Of the 92 patients whose Gd-MRI-based ATVA was measured, 20 were excluded from the final analysis because of inconclusive α_exit_ values (>120° and <140°) likely due to insufficient image quality from the Gd-MRI data.

### Reproducibility of the ATVA Measurements

Internal consistency of the Gd-MRI-based angle measurements and patient endotyping was tested by two independent investigators (AE and DB) who reassessed the Gd-MRI data from a randomly selected 14% of the cases. The investigators were blinded to the previous measurements when remeasuring α_exit_ and assigning cases to the endotype subgroups per the procedures described above. The interobserver test-retest reliability of the endotype assignments (MD-dg vs. MD-hp) was determined using Cohen's kappa coefficient (x). A x-value of 0.78 was determined, indicating excellent test-retest agreement per (13).

### Clinical Data Collection and Processing

Clinical records, audiometric and vestibular test data, and radiology reports were reviewed by two investigators (CB and TH), who were blinded to the original imaging data and endotyping results. All data were collected in a structured digital database with predefined variables and categories. After collecting the data, all variables in the database were checked for categories with excessively low cell frequencies (<4). Those categories were merged with others from the same variable based on clinical meaningfulness to retain sufficient statistical power.

### Statistical Analysis

Null hypotheses were formulated and statistical tests were selected before collecting the data. Second-step statistical analysis (*post hoc* testing) was clearly indicated (see respective paragraph). For binary variables, a chi-square test was performed if all category frequencies were greater than five; otherwise, Fisher's exact test was performed. Continuous variables were analyzed using a two-tailed Student's *t*-test for independent samples if the data were normally distributed; otherwise, a two-tailed Mann-Whitney U-test was performed. If not otherwise specified, statistical analyses were performed using IBM SPSS Statistics, version 24 (IBM Corp., Armonk, NY, USA). *P*-values were categorized into levels of evidence against H_0_ (very strong evidence, *p* < 0.001; strong evidence, *p* = 0.001–0.01; evidence, *p* = 0.01–0.05; weak evidence, *p* = 0.05–0.1; and little or no evidence, *p* > 0.1) per (14).

### Multiple Testing Correction

Twenty-eight independent null hypotheses were tested on the sample data. (A Benjamini–Hochberg procedure for multiple comparisons was used to control the false discovery rate (FDR) (15) see also the following paragraph). Subsequent *p*-values are followed by a statement regarding their significance per the Benjamini-Hochberg procedure (significant [sig.] or not significant [n. s.]) (16).

### Rationale for Choosing the FDR's Critical Value

This study's purpose was to exploratively and exhaustively screen for clinical variables that differed between the two predefined patient subgroups and that would potentially contribute to the variable's clinical meaningfulness. Therefore, in our statistical analysis, we intended to minimize the risk of false negatives (type II error) while retaining an appropriate risk of false positives (type I error), which was controlled by the FDR (15). In contrast to large-scale multiple-testing studies (e.g., genomics studies with >10^3^ null hypotheses) that typically apply an FDR of 0.05 to minimize the type I error probability, we chose an FDR of 0.15. This value is associated with a reduced type II error rate and is commonly used in explorative and proof-of-concept studies (17, 18) in which not overlooking any true-positive results is prioritized.

### *Post hoc* Analysis

In all contingency tables where significant differences were detected (based on an FDR of 0.15) between the groups, we performed *post hoc* testing per (19), enabling rigorous control of type I error rates: all three possible pairwise comparisons were performed between one variable and the other two, followed by a Bonferroni correction (in which the *p*-value is divided by the number of tests).

### Longitudinal Analysis

To investigate whether the two MD endotypes differed in hearing loss progression over time (i.e., the slope of pure-tone averages [PTAs] respective of time), we performed a longitudinal analysis on data from each patient's repeated audiometric measurements. In bilateral cases, each ear was considered a separate case. A linear mixed-effects model was used with a subject-specific random intercept and a random slope for time. This model enabled varying baseline measurements and time-varying trends among subjects, considering data imbalance and unequal time-spacing. PTA measurements constituted the response, while time and endotype were the explanatory variables. Moreover, an interaction term between pathology type and time was included in the model to allow a pathology-specific time trend. The longitudinal analysis was performed in the R programming language using the function, *lmer()*, from the analysis-specific package, *lme4* (20, 21).

### Volume Reconstructions

**Figure 3:**
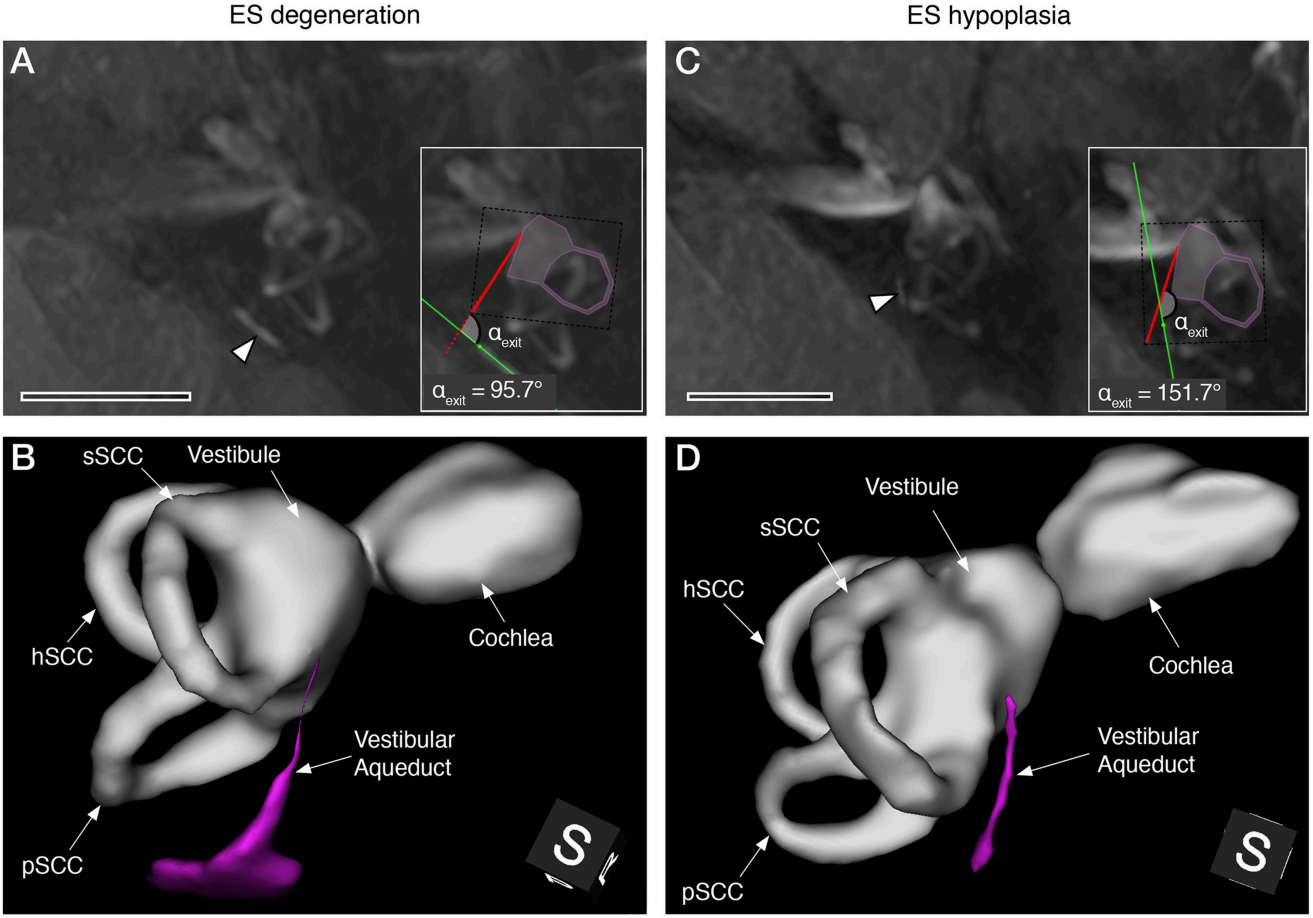
Gd-MRI VA morphology in representative MD cases with degenerative **(A,B)** and hypoplastic **(C,D)** ES pathology. Maximum intensity projections of axial plane images **(A,C)**, and 3D reconstructions (postero-superior view) of the labyrinthine fluid spaces with the VA shown in magenta **(B,D)**. Arrowheads in **(A,C)** indicate the VA in the opercular region. Insets in **(A,C)** show angle measurements of the VA's angular trajectory as described previously (7) (sSCC, superior semicircular canal; hSCC, lateral semicircular canal; pSCC, posterior semicircular canal). Scale bars, 10 mm.

Inner ear volumes (Figure 3) were reconstructed from Gd-MRI data using 3D Slicer software [version 4.8.1 for Mac OS X (22)].

## Results

### Patient Endotyping and Baseline Characteristics

Of the 72 patients who were ultimately included, 55 (76.4%) demonstrated angles (α_exit_) ≤120° in the clinically affected inner ear(s). This subgroup was histopathologically diagnosed with ES degeneration (6) and is hereafter referred to as the MD-dg subgroup (Figures 3A,B). In 17 patients (23.6%), angles (α_exit_) ≥140° were observed, corresponding to a histopathological diagnosis of ES hypoplasia [(7); MD-hp subgroup; Figures 3C,D]. Table 1 shows the 72 endotyped patients' baseline characteristics.

**Table 1:** Key baseline characteristics of the study sample.

	**Total MD patients included (*n* = 72)**
Female:male ratio	41 (56.9%): 31 (43.1%)
Unilateral:bilateral ratio	64 (88.9%): 8 (11.1%)
Disease duration (^*^)	10.1 (6.1) years
Age at first MD symptoms	46.8 (12.0) years
Age at inclusion	56.8 (12.7) years

*Mean (SD) or absolute numbers (percentage)*.

* *Mean disease durations at inclusion were similar in both subgroups (MD-dg: 9.8 ± 6.4 years MD-hp: 10.6 ± 5.1 years; p = 0.685)*.

### Between-Endotype Clinical Differences

**Figure 4:**
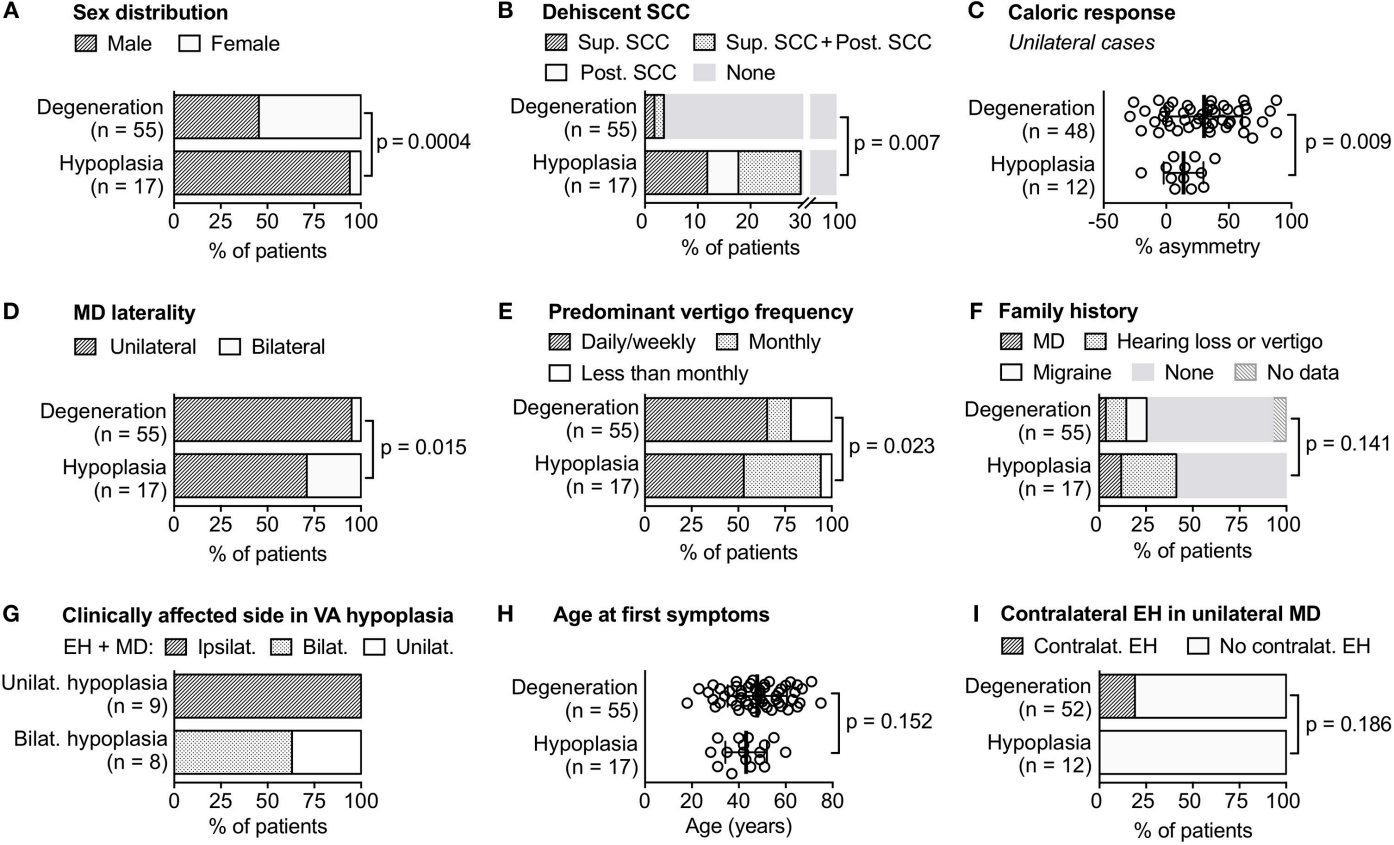
Clinical differences between and within the two MD endotypes. Data from **(A–F,H)** are also shown in Table 2, data from **(G)** and **(I)** were not part of the initial null hypotheses. Bilat, bilateral; contralat, contralateral; EH, endolymphatic hydrops; ipsilat, ipsilateral; MD, Meniere's disease; post, posterior; SCC, semicircular canal; sup, superior; unilat, unilateral; VA, vestibular aqueduct.

From 28 clinical variables (Table 2), seven variables showed evidence to very strong evidence (14) for group differences (MD-dg vs. MD-hp endotypes), which are highlighted together with other potentially clinically meaningful variables (Figure 4).

**Table 2:** Demographic and clinical characteristics tested for between-group differences.

**Variable**	**Degeneration (*n* = 55)**	**Hypoplasia (*n* = 17)**	**Difference (95% CI)**	***p*-value**	**Benjamini-Hochberg significance**
Female:male ratio	30 (54.5%): 25 (45.5%)	1 (5.9%): 16 (94.1%)	n. a.	0.0004	sig.
Headache type					
- Migraineous	20 (36.4%)	4 (23.5%)			
- Non-migraineous	5 (9.1%)	8 (47.1%)	n. a.	0.004	sig.
- None	30 (54.5%)	5 (29.4%)			
SCC dehiscence on affected side	2 (3.6%)	5 (29.4%)	n. a.	0.007	sig.
Caloric response asymmetry (unilateral cases)—% asymmetry	30.2 ± 30.4 (*n* = 48)	13.5 ± 15.2 (*n* = 12)	16.7 (4.4 to 29.1)	0.009	sig.
Laterality					
- Unilateral	52 (94.5%)	12 (70.6%)	n. a.	0.015	sig.
- Bilateral	3 (5.5%)	5 (29.4%)			
Vertigo frequency					
- Daily/weekly	36 (65.5%)	9 (52.9%)			
- Monthly	7 (12.7%)	7 (41.2%)	n. a.	0.023	sig.
- Other	12 (21.8%)	1 (5.9%)			
Family history					
- MD	2 (3.6%)	2 (11.8%)			
- Hearing loss/vertigo	6 (10.9%)	5 (29.4%)			
- Migraine	6 (10.9%)	0 (0.0%)	n. a.	0.141	n. s.
- None	37 (67.3%)	10 (58.8%)			
- No data	4 (7.3%)	0 (0.0%)			
Age at first symptoms	47.9 ± 12.6	43.1 ± 8.9	4.8 (−0.8 to 10.3)	0.152	n. s.
Cardiovascular comorbidities	20 (36.4%)	3 (17.6%)	n. a.	0.234	n. s.
Hearing aid(s)	20 (36.4%)	9 (52.9%)	n. a.	0.265	n. s.
Vegetative symptoms	47 (85.5%)	12 (70.6%)	n. a.	0.277	n. s.
Hydrops grade—median (25th−75th percentiles)	1.3 (1.0 to 2.0)	1.0 (0.9 to 1.5)	n. a.	0.289	n. s.
oVEMP asymmetry ratio	6.5 ± 24.3 (*n* = 41)	13.0 ± 19.4 (*n* = 13)	6.5 (−7.1 to 20.0)	0.324	n. s.
cVEMP asymmetry ratio	6.9 ± 22.4 (*n* = 38)	15.8 ± 22.8 (*n* = 13)	8.9 (−0.5 to 14.2)	0.333	n. s.
Dynamic visual acuity loss (logMAR)—median (25th−75th percentiles)	0.4 (0.5 to 0.6) (*n* = 40)	0.3 (0.4 to 0.6) (*n* = 7)	n. a.	0.349	n. s.
Hearing loss (last PTA)—median (25th−75th percentiles)	40.0 (19.9 to 54.6)	42.1 (26.8 to 42.1)	n. a.	0.403	n. s.
Head impulse test gain ratios—median (25th−75th percentiles)	1.1 (0.9 to 1.3) (*n* = 49)	1.0 (0.8 to 1.3) (*n* = 9)	n. a.	0.410	n. s.
Neck or spine problems	9 (16.4%)	1 (5.9%)	n. a.	0.434	n. s.
Hearing loss (first PTA)—median (25th−75th percentiles)	28.3 (10.8 to 45.5)	28.25 (19.3 to 44.4)	n. a.	0.443	n. s.
Maximal MD therapy					
- Betahistine/cinnarizine	17 (30.9%)	6 (35.3%)			
- Intratympanic dexamethasone/lidocaine	34 (61.8%)	11 (64.7%)	n. a.	0.665	n. s.
- Intratympanic gentamicin	4 (7.3%)	0 (0.0%)			
Photo-/phono-phobia	17 (30.9%)	6 (35.3%)	n. a.	0.735	n. s.
Hearing loss pattern (first PTA)					
- Low	6 (10.9%)	0 (0.0%)			
- High and low (peak)	25 (45.5%)	9 (52.9%)			
- High/other	9 (16.4%)	3 (17.6%)	n. a.	0.737	n. s.
- All frequencies	13 (23.6%)	5 (29.4%)			
- No hearing loss	2 (3.6%)	0 (0.0%)			
Migraineous aura	11 (20.0%)	3 (17.6%)	n. a.	0.830	n. s.
Maximal migraine therapy					
- Magnesium, vitamin B2, flunarizine	13 (23.7%)	4 (23.5%)			
- Valproate, triptans	10 (18.1%)	4 (23.5%)	n. a.	0.928	n. s.
- None	32 (58.2%)	9 (52.9%)			
Hearing loss pattern (last PTA)					
- Low	1 (1.8%)	0 (0.0%)			
- High and low	17 (30.9%)	6 (35.3%)			
- High/other	10 (18.2%)	2 (11.8%)	n. a.	0.929	n. s.
- All frequencies	23 (41.8%)	8 (47.1%)			
- No hearing loss	3 (5.5%)	0 (0.0%)			
- No data	1 (1.8%)	1 (5.9%)			
First MD symptom					
- Hearing loss	13 (23.6%)	4 (23.5%)			
- Vertigo	11 (20.0%)	5 (29.4%)			
- Tinnitus/aural fullness	5 (9.1%)	1 (5.9%)	n. a.	0.929	n. s.
- Multiple	21 (38.2%)	7 (41.2%)			
- No data	5 (9.1%)	0 (0.0%)			
Allergies	6 (10.9%)	2 (11.8%)	n. a.	0.992	n. s.
Vertigo quality					
- Rocking	12 (21.8%)	4 (23.5%)			
- Spinning	41 (74.6%)	13 (76.5%)	n. a.	1.000	n. s.
- Other	2 (3.6%)	0 (0.0%)			

*Values are reported as absolute numbers (percentage), mean ± SD or median with 25th and 75th percentiles. The number in brackets indicates the number of patients included in the analysis if some patients in the group were excluded. 95% CI, 95% confidence interval; cVEMP/oVEMP, cervical/ocular vestibular-evoked myogenic potentials; logMAR, logarithm of minimum angle of resolution; MD, Meniere's disease; n. a., not applicable; n. s., not significant; PTA1, first available pure-tone audiometry; PTA4, last available pure-tone audiometry; SCC, semicircular canal; sig., significant*.

Very strong evidence was found for a different sex distribution (*p* = 0.0004, sig.; Figure 4A), with a strong preponderance of male patients in the MD-hp group (male-to-female ratio = 1:0.06) and a nearly balanced male-to-female ratio in the MD-dg group (1:1.2). The Gd-MRI data strongly evidenced radiological signs of SCC dehiscence (Figure 4B; i.e., no clear spatial separation of signals from the superior, posterior or both SCCs and the cerebrospinal fluid space) more often in the MD-hp group (5/17 [29.4%]) than in the MD-dg group [2/55 [3.6%]; *p* = 0.007, sig.]. The objective vestibular test data strongly evidenced, on average, greater reductions of bithermal caloric responses (Figure 4C) on the clinically affected side in the MD-dg group (30.2 ± 30.4% asymmetry, *n* = 48) than in the MD-hp group (13.5 ± 15.2% asymmetry, *n* = 12; *p* = 0.009, sig.). Notably, there was no evidence that the mean age at caloric testing differed between groups (MD-dg group: 54.9 ± 13.5 years; MD-hp group 52.4 ± 8.7 years, *p* = 0.38). We found further evidence for a higher frequency of bilateral clinical manifestations (Figure 4D) in the MD-hp group (5/17 [29.4%]) than in the MD-dg group (3/55 [5.5%]; *p* = 0.015, sig.), as well as for different frequency patterns of vertigo symptoms (Figure 4E) between groups (*p* = 0.023, sig.). *Post hoc* comparisons (Table 3) evidenced a group difference when testing the frequency pattern “monthly” (MD-dg: 12.7%, MD-hp: 41.2% of patients) vs. the other two frequency patterns (“daily/weekly” and “less than once per month”; *p* = 0.048, Bonferroni corrected). Although the initial analysis revealed no evidence of group differences regarding a positive family history for MD, non-age-related hearing loss or vertigo and migraine (Figure 4F), *post hoc* analysis for MD and hearing loss/vertigo symptoms only (Table 3) was more prevalent in relatives of MD-hp patients (41.2%; MD-dg group: 15.7%, data available from *n* = 51; *p* = 0.028, Bonferroni corrected).

**Table 3:** *Post-hoc* tests.

		**ES pathology**		
**Clinical feature**	**Category**	**Degeneration**	**Hypoplasia**	***p*-value (Bonferroni corrected)**
Vertigo	Monthly	7 (12.7%)	7 (41.2%)	*p* = 0.048
	Daily/weekly or less than once per month	48 (87.3%)	10 (58.8%)	
Headache type (males)	Migraineous	6	4	*p* = 0.211
	Non-migraineous	5	7	
	Other	14	5	
Family history	MD, hearing loss and/or vertigo	8 (15.7%)	7 (41.2%)	*p* = 0.028
	No family history	43 (84.3%)	10 (58.8%)	

For the remaining variables, no evidence of group differences was found, or no statistical analysis was performed. Those variables nonetheless represent clinically meaningful and potentially endotype-segregating features (see Discussion section). Although strong evidence suggested that headache symptom frequencies differed between the two groups in the primary statistical analysis (*p* = 0.004, sig., Table 2), *post hoc* analysis (Table 3) revealed no evidence of a group difference when only male patients in both groups were compared to eliminate sex as a confounding factor [higher prevalence of migraine headaches in women than in men in the general population (23)]. In the MD-hp group, unilateral hypoplastic ES pathology was invariably associated with ipsilateral endolymphatic hydrops and ipsilateral MD. However, among the eight patients with bilateral ES hypoplasia, five demonstrated endolymphatic hydrops and MD symptoms in both inner ears, while three had only one ear affected (Figure 4G). The mean age when MD symptoms first manifested was slightly earlier in the MD-hp group (43.1 ± 8.9 years) than in the MD-dg group (47.9 ± 12.6 years; *p* = 0.152, n. s.; Figure 4H). Endolymphatic hydrops without evidence of clinical affection [i.e., “clinically silent” endolymphatic hydrops (8, 24)] was exclusively observed in the contralateral inner ears from unilaterally affected MD-dg patients (19.2% of patients; *p* = 0.186, n. s.; Figure 4I).

## Discussion

The following sections discuss the endotype-specific clinical characteristics and their potential implications for clinically managing MD patients (synopsis in Table 4).

**Table 4:** Endotype-specific clinical considerations.

**Endotype (patient subgroup)**	**Laterality**	**Endotype frequency**	**Characteristics/considerations**
ES hypoplasia (MD-hp patients)	Overall	23.6%	Specific radiological feature (VA bending angle ≥140°) Prognostic radiological screening possible Possible concomitant SSC dehiscence syndrome ES not visualizable for surgical ES enhancement/shunting (^*^)
	Unilateral	16.7%	Very low risk of developing bilateral MD (†) Most suitable for vestibular ablative therapy
	Bilateral	6.9%	Very high risk of developing bilateral MD Avoid performing vestibular ablative therapy (‡) Significant impact on auditory/vestibular function and quality of life must be expected
ES degeneration (MD-dg patients)	Overall	76.4%	Suitable for ES surgery if considered (§) Consider average higher frequency of vertigo symptoms, more severe vestibular hypofunction when establishing treatment Consider sequential imaging to rule out ES tumors if symptomatology increases over baseline fluctuations (||)
	Unilateral	72.2%	“Clinically silent” contralateral EH (19.2%) Unknown risk for progression to bilateral disease Increased risk to perform vestibular ablative treatment
	Bilateral	4.2%	Least frequent endotype, 4.2% of patients

* *Considering the anticipated low chance of intraoperatively identifying the operculum (25)*.

(†) *equal to the overall risk of developing MD with degenerative ES pathology in the general population [i.e., 0.15% based on the MD prevalence of 0.2% (26) and a prevalence of degenerative ES pathology of 76.4% among MD patients in the present study]*.

(‡) *upon initial presentation with clinically unilateral symptoms*.

(§) *due to degenerative changes of extraosseous ES in these patients (6), we consider the rationale for ES surgery questionable. (||) according to Kirsh et al. (27). In MD-hp patients, ES hypoplasia sufficiently explains the etiology*.

### Endotype “ES Hypoplasia” (MD-hp Subgroup)

ES hypoplasia was present in 23.6% of our cohort. Unilateral ES hypoplasia was invariably associated with ipsilateral endolymphatic hydrops and MD but not with any radiological/clinical signs for contralateral affection. In those cases, the risk of developing MD in the contralateral inner ear (normal VA angle) is probably low, most likely comparable to the risk of MD with an ES degenerative endotype in the general population [i.e., ~0.15% per a calculation based on data from this study and (26)], and they may be suitable candidates for ablative therapies [e.g., gentamicin injections) in the affected ear if clinically indicated. MD-hp patients should be considered with caution for ES surgical procedures [decompression, shunting, or endolymphatic duct blockage; reviewed in (28, 29)] since the hypoplastic operculum of the VA is considerably smaller than normal, and the extraosseous ES portion is mostly absent (6). A recent retrospective study showed that during ES surgery in MD patients, intraoperatively visualizing the operculum was impossible in 28% of the cases (25). This number very closely matches the hypoplastic ES-endotype prevalence found in the present study (23.6%). In almost all MD-hp patients, bilateral ES hypoplasia was associated with bilateral endolymphatic hydrops and MD; however, three of those patients showed only unilateral hydrops and clinical symptoms at the time of data collection (Figure 4G). These three patients are expected to develop bilateral MD later in life based on the consistent association between ES hypoplasia and clinical symptoms that we found previously in post-mortem cases (6, 7). Thus, in all MD-hp patients with bilateral ES hypoplasia, a bilaterally impaired audiovestibular function (bilateral MD) with a significantly impacted quality of life should be expected, and those patients should be considered with caution for ablative therapies. Furthermore, MD-hp patients may have an increased risk of concomitant audiovestibular symptoms caused by SCC dehiscence syndrome, as suggested by the higher prevalence (29.4%) of suspected (posterior) SCC dehiscence on the MRI data. In contrast, SCC dehiscence was suspected radiologically in only 3.6% of MD-dg patients, mostly of the superior SCC, which is comparable to the reported overall prevalence of superior SCC dehiscence in the general population [3–10% (30–32)]. The epidemiological features associated with this endotype (predominantly men, familial clustering of MD, and tendency toward earlier disease onset) support the hypothesis of a genetic/developmental cause. Therefore, the endotype's specific radiological feature (VA bending angle ≥140°) may be useful for predictive clinical screenings (e.g., in family members of MD-hp patients) for this potentially inheritable inner ear pathology and to prognosticate the risk of developing MD.

### Endotype “ES Degeneration” (MD-dg Subgroup)

ES degeneration was observed in 76.4% (*n* = 55; MD-dg group) of patients. This endotype (VA angle ≤120°) is radiologically diagnosed by excluding the hypoplastic endotype (VA angle ≥140°), but it cannot be distinguished from a normal ES (VA angle ≤120°) using the Gd-MRI-based criteria described herein. Thus, in contrast to the hypoplastic endotype, no potential prognostic marker for future bilateral manifestation is available to date. This endotype was exclusively associated with contralateral “clinically silent” endolymphatic hydrops (i.e., hydrops without clinical signs of inner ear dysfunction, particularly without MD). Clinically silent hydrops was observed in 19.2% of all unilateral MD-dg patients, which is consistent with previously reported numbers from MRI studies investigating unilaterally affected MD patients [16.0–23.3% (8, 33)]. Since clinically silent hydrops reportedly progresses to MD in 33% of cases (34), MD-dg patients with contralateral hydrops may have an elevated risk of developing bilateral MD and should be considered for ablative therapy with caution. However, MD-dg patients may be generally considered suitable candidates for ES surgery (decompression or shunting) since the operculum, which exhibits normal anatomy in these cases, allows its reliable intraoperative visualization. However, per our previous human pathology study (6), the extraosseous ES portion is degenerating in these cases, which calls into question the proposed mode of function of these treatments. On average, a higher frequency of vertigo attacks and a more severely impaired vestibular (caloric) function was associated with this endotype. These findings may be explained by a supposedly more sudden manifestation of ES degeneration in the matured inner ear, as opposed to the developmentally inherent ES hypoplasia. ES degeneration therefore probably has a more disruptive effect on vestibular function due to the adult inner ear's limited capability to develop compensating homeostatic mechanisms. In contrast in the developing inner ear, in the presence of ES hypoplasia, such compensatory function may be established more efficiently.

### Between-Endotype Comparisons With Little or No Evidence for Group Differences

Of the 28 tested clinical variables, we found weak to no evidence for endotype differences for 21 variables (Table 2) and for a longitudinal analysis of hearing loss progression (Supplementary Figure 1; Benjamini-Hochberg significance: n. s.). However, our retrospective explorative approach does not categorically rule out those 21 variables as clinically meaningful, endotype-segregating features. Specifically, variables such as migraine headache prevalence, age at first MD symptom onset, and prevalence of “clinically silent” contralateral endolymphatic hydrops should, in our view, remain of interest in future endotyping studies.

Although the migraine headache prevalence did not differ between endotypes, the overall prevalence of migraine symptoms in our cohort (59.9%) was similar to that of a previous study [56% (35)] and considerably higher than the prevalence in the general population [11–18% (36–38)]. Future studies should more specifically address questions such as whether migraines are associated—or even share a common etiopathology—with a distinct MD endotype.

An earlier age of onset of the first MD symptoms in MD-hp patients was evident in our previous temporal bone study (6) (MD-hp: 37.7 ± 19.0 years; MD-dg: 58.1 ± 20.5 years; *p* = 0.038) but not in the present study (MD-hp: 43.1 ± 8.9 years; MD-dg: 47.9 ± 12.6; *p* = 0.152, n. s.). An earlier symptom onset in the MD-hp group (although not evident in the present study) could be explained by the likely prenatally determined hypoplastic ES pathology (7), which presumably renders the inner ear susceptible to precipitating factors that ultimately cause MD. In contrast, ES degeneration in MD-dg patients is thought to be an acquired pathology, possibly associated with age-related factors, and therefore may explain MD's tendency to manifest later in life.

A “clinically silent” (no MD symptoms) endolymphatic hydrops is a frequent [5–26%, (8, 24, 39)] histological and radiological entity in the contralateral ears from unilaterally affected Meniere's patients, and was in the present study found exclusively in the MD-dg endotype (19.2%; *p* = 0.186, n. s.). The frequent association of this endotype with bilateral hydrops may suggest limited or still progressing ES degeneration as the cause for contralateral, clinically silent hydrops, and further suggests a systemic (e.g. immune-mediated or vascular) etiology of degenerative ES pathology. Although progression to bilateral MD was rarely observed in the MD-dg group (4.2%); longer prospective observations are required to determine whether clinically silent hydrops represents a precursor lesion of MD.

### Patient Subgrouping Approaches in MD

Patient stratification algorithms in MD can be divided into phenomenology (clinical features)-based “top-down” algorithms, and etiology (endotype)-based ”bottom-up” algorithms. Frejo et al. used disease laterality (uni vs. bilateral) as a principal group classifier, and, in a second step, applied data mining procedures (cluster analysis) to identify clinical features (autoimmune disorders, migraine, MD family history, synchronic vs. metachronic onset of hearing loss) that segregated five discrete subgroups of uni and bilateral MD patients, respectively (40, 41). In a follow-up study, Frejo et al. then used an etiology-based approach and distinguished two subgroups with different cytokine profiles among uni and bilateral MD patients (42). They suggested an immune-mediated etiology in the subgroup that exhibited elevated interleucin-1β plasma levels as well as mold-induced TNF-α secretion. Notably, some subgroups defined by Frejo et al. share certain group-defining features with the MD-dg and MD-hp endotypes. Further studies that combine the described subgrouping/endotyping algorithms will determine whether the subgroups defined by the different approaches truly overlap.

### Limitations of the Study

The overall study design was retrospective and exploratory. Thus, for statistical between-group comparisons, an FDR was chosen that allowed us to identify many endotype-specific clinical features. Moreover, few patients (*n* = 72) were available for this study. Consequently, the statistical power for some group comparisons was low, and the results from this investigation should be verified in a larger prospective cohort study.

For the 20 excluded patients, α_exit_ angles between 120 and 140° were measured, or the VA could not be visualized (Figure 1). Since such α_exit_ values or non-visualization of the VA were lacking in a previous retrospective MD case series (*n* = 64) in which HRCT temporal bone data were measured (7), we consider this a problem of insufficient Gd-MRI resolution, likely due to movement artifacts in individual datasets.

For most included patients, the initial diagnostic test data (PTA, vestibular diagnostics) were acquired at the time of diagnosis, whereas for a minority of patients, these initial tests were performed several months or years after being diagnosed. This inhomogeneity of the available data is a confounding factor, particularly in the longitudinal analysis of hearing thresholds (Supplementary Figure 1).

## Conclusion

This novel endotype-driven diagnostic approach allowed distinguishing clinically meaningful MD patient subgroups with different etiologies and phenotypes. This approach may (i) help select more homogeneous patient cohorts for future clinical studies, (ii) refine clinical diagnoses, (iii) improve clinical decision-making, and (iv) help prognosticate the disease course in individual patients. The results from this retrospective study require validation in prospective cohort studies.

## Author Contributions

AE: study conception and design; DB, CB, TH, and AE: data acquisition; DB, CB, TH, EM, SM, and AE: data analysis and interpretation; DB and AE: drafted the manuscript; AM, VW, SM, and BS: critically reviewed and revised the manuscript.

### Conflict of Interest Statement

The authors declare that the research was conducted in the absence of any commercial or financial relationships that could be construed as a potential conflict of interest.
