# Supplementary material for: Endotype-Phenotype Patterns in Meniere's Disease Based on Gadolinium-Enhanced MRI of the Vestibular Aqueduct
Source: Front Neurol. 2019 Apr 5;10:303. doi: 10.3389/fneur.2019.00303 (PMC6459933; doi:10.3389/fneur.2019.00303)
Supplement: Supplementary file 1 [file Data_Sheet_1.docx]

Supplementary Material

Endotype-Phenotype Patterns in Meniere’s Disease Based on Gadolinium-Enhanced MRI of the Vestibular Aqueduct

**David Bächinger^1,2^, Catrin Brühlmann^2^, Tim Honegger^2^, Eleftheria Michalopoulou^3^, Arianne Monge Naldi^4^, Vincent G. Wettstein^1,2^, Stefanie Muff^3^, Bernhard Schuknecht^5^, Andreas H. Eckhard^1,2^,***

^1^ Department of Otorhinolaryngology, Head and Neck Surgery, University Hospital Zurich

^2^ University of Zurich, Zurich, Switzerland

^3^ Institute for Epidemiology, Biostatistics, and Prevention, Department of Biostatistics, University of Zurich, Zurich, Switzerland

^4^ Department of Otorhinolaryngology, University Children's Hospital Zurich, Zurich, Switzerland

^5^ Medical Radiological Institute MRI, Zurich, Switzerland

***** **Correspondence:** Andreas H. Eckhard, Department of Otorhinolaryngology, University Hospital Zurich, Frauenklinikstrasse 24, 8091 Zurich, Switzerland; Email: [AndreasHeinrich.Eckhard@usz.ch](mailto:AndreasHeinrich.Eckhard@usz.ch)

# Supplementary Figures and Tables

**Supplementary Figure 1:** Longitudinal analysis of hearing with estimated fixed effects of endotype, time and their interaction. In 8 bilateral cases, each ear was considered a separate case, resulting in a total of 80 ears analyzed (58 MD-dg, 22 MD-hp). The MD-dg group was chosen as the reference group. **A** In the MD-dg group, the baseline pure-tone average was 29.7 dB (95 % Wald confidence interval 25.0 dB to 34.4 dB). In the MD-hp group, the baseline pure-tone average was 1.7 dB higher (95 % Wald confidence interval -7.3 dB to 10.8 dB, p = 0.71). In the MD-dg group, hearing decreased by an average of 2.1 dB (1.2 dB to 3.0 dB, p < 0.0001) per year, which provides strong evidence for an effect of time on the course of hearing loss. -However, there was no evidence that the course of hearing loss differed between the two subgroups (difference between endotype slopes 1.8 dB, 95 % Wald confidence interval -1.9 dB to 1.3 dB, p = 0.68). **B** Individual trajectories of the pure-tone average measurements over time. The baseline measurement of each subject is subtracted from the rest of the measurements of the same subject.
